# Case Report: Soft tissue infection with *Burkholderia thailandensis* capsular variant: case report from the Lao PDR

**DOI:** 10.12688/wellcomeopenres.22706.1

**Published:** 2024-08-01

**Authors:** Souphaphone Vannachone, Manophab Luangraj, David Dance, Narisara Chantratita, Natnaree Saiprom, Rathanin Seng, Sarunporn Tandhavanant, Sayaphet Rattanavong, Andrew Simpson, Tamalee Roberts

**Affiliations:** 1 Lao-Oxford-Mahosot Hospital Wellcome Trust Research Unit, Vientiane, Vientiane Capital, 0100, Lao People's Democratic Republic; 2 Nuffield Department of Medicine, Centre for Tropical Medicine & Global Health, University of Oxford, Oxford, UK; 3 Faculty of Infectious and Tropical Diseases, London School of Hygiene and Tropical Medicine, London, UK; 4 Department of Microbiology and Immunology, Faculty of Tropical Medicine, Mahidol University, Bangkok, Thailand; 5 Faculty of Tropical Medicine, Mahidol University, Mahidol-Oxford Tropical Medicine Research Unit, Bangkok, Thailand

**Keywords:** Burkholderia thailandensis, B. thailandensis capsular variant, Lao PDR

## Abstract

**Background:**

*Burkholderia thailandensis* is an environmental bacteria closely related to *Burkholderia pseudomallei* that rarely causes infection in humans. Some environmental isolates have shown to express a capsular polysaccharide known as *B. thailandensis* capsular variant (BTCV), but human infection has not previously been reported. Although *B. thailandednisis* has been identified in environmental samples in Laos before, there have not been any human cases reported.

**Case:**

A 44-year-old man presented to a district hospital in Laos with a short history of fever and pain in his left foot. Physical examination identified a deep soft-tissue abscess in his left foot and an elevated white blood count. A deep pus sample was taken and melioidosis was suspected from preliminary laboratory tests. The patient was initially started on cloxacillin, ceftriaxone and metronidazole, and was then changed to ceftazidime treatment following local melioidosis treatment guidelines.

**Laboratory methods:**

A deep pus sample was sent to Mahosot Hospital microbiology laboratory where a mixed infection was identified including *Burkholderia* sp. Conventional identification tests and API 20NE were inconclusive, and the *B. pseudomallei*-specific latex agglutination was positive. The isolate then underwent a *Burkholderia* species specific PCR which identified the isolate as *B. thailandensis.* The isolate was sent for sequencing on the Illumina NovaSeq 6000 system and multi-locus sequence typing analysis identified the isolate had the same sequence type (ST696) as *B. thailandensis* E555, a strain which expresses a *B. pseudomallei*-like capsular polysaccharide.

**Conclusion:**

This is the first report of human infection with *B. thailandensis* in Laos, and the first report of any human infection with the *B. thailandensis* capsular variant. Due to the potential for laboratory tests to incorrectly identify this bacteria, staff in endemic areas for *B. thailandensis* and *B. pseudomallei* should be aware and ensure that appropriate confirmatory methods are used to differentiate between the species.

## Plain Language Summary

> *Burkholderia thailandensis* is a bacteria that is found in the environment. Rarely, this bacteria can cause infection in humans. Here we report a *B. thailandensis* infection in a 44 year old male in Laos. The patient sustained a puncture wound in his left foot and when presenting at a district hospital was prescribed cloxacillin. The wound did not improve and on day three of admission, a pus sample was sent to Mahosot Hospital Microbiology Laboratory for investigation. A preliminary diagnosis of melioidosis, caused by the bacteria *Burkholderia pseudomallei,* was made and antibiotic treatment was changed. Additional laboratory investigation determined that the isolate was actually *B. thailandensis* and antibiotic treatment was further changed. Due to the inconclusive results of the initial laboratory tests, the isolate was sent for sequencing and was identified as a strain which expresses a *B. pseudomallei*-like capsular polysaccharide. This is the first report of infection with *B. thailandensis* in Laos and the first report of infection with a *B. thailandensis* capsular variant.

## Introduction

*Burkholderia thailandensis* is closely related to *Burkholderia pseudomallei,* the causative agent of melioidosis, a potentially fatal infectious disease found in Southeast Asia and Northern Australia ^1^. *B. thailandensis* lives in soil and is generally considered non-pathogenic, in comparison to *B. pseudomallei* ^2, 3^. There have, however, been several reports of human infection with *B. thailandensis*, ranging from localized to life-threatening infection ^4, 5^. Some environmental isolates of *B. thailandensis*, including one from Lao PDR (Laos), have been shown to express a capsular polysaccharide that cross-reacts with that of *B. pseudomallei*, known as *B. thailandensis* capsular variants (BTCV), but as yet infection with such a strain has not been reported ^6^. In Laos, there have been more than 1000 culture confirmed melioidosis cases in the past two decades since the first report in 1999 ^7^ but there have been no reports of *B. thailandensis* infection. We report here the first case of soft tissue infection caused by a mixture of bacteria including BTCV that occurred in Vientiane Province, Laos.

## Case report

On 9 ^th^ January 2019, a 44 year old man was admitted to a local district hospital with a short history of fever and pain in his left foot. The patient was a farmer from Vientiane Province, Laos, and lived approximately 60 kilometers from Vientiane Capital. He was previously considered fit and healthy. Two days prior to presenting to hospital, he suffered a puncture wound to his left foot while cleaning out a fish pond on his property. He started having pain that afternoon and the area started to swell. The next day he went to work in a rice field and self-treated with antibiotics bought from a local pharmacy, however his symptoms did not improve. On admission to hospital, his physical examination identified a deep soft-tissue abscess in his left foot. The patient’s white blood count was elevated at 16.3 × 10 ^9^ cells/L, with a markedly elevated granulocyte count (98.1%) and blood glucose was normal (125 mg/dL). The patient was treated with intravenous (IV) cloxacillin 1 g four times per day. On day three of admission (11 ^th^ Jan), the left foot abscess was incised and drained; a deep pus sample was sent to the Microbiology Laboratory at Mahosot Hospital, Vientiane. The same day, the patient developed a high-grade fever and worsening of the abscess on the left ankle with severe pain on the left foot and loss of ability to walk. Ceftriaxone (2 g/day) and metronidazole (500 g 8 hourly) were added and cloxacillin discontinued, along with appropriate wound debridement, dressing and cleaning with normal saline, although without improvement. On day eight, haemoculture, urine and throat swab cultures were collected and sent to the Mahosot Hospital Microbiology Laboratory, as melioidosis was suspected, based on preliminary pus culture results. *B. pseudomallei* was subsequently not isolated from any sample, but treatment was changed to ceftazidime (2 g three times per day) as per local melioidosis treatment guidelines. On 22 ^nd^ January IV treatment was discontinued, the patient was discharged well and with one week of co-trimoxazole oral treatment to complete at home after discharge.

The foot abscess pus was cultured on blood agar (Oxoid, incubated in 5–10% CO _2_ for 48 h) and Ashdown agar (made in-house, incubated in air and read daily for 4 days). Direct Gram staining demonstrated Gram-positive cocci in chains and Gram-negative coccobacilli. No acid-fast bacilli were seen in the Ziehl Neelsen stain. Culture yielded a mixed growth of five organisms which were identified using standard biochemical and API tests (bioMerieux, Marcy L’Etoile, France). Comprehensive antimicrobial susceptibility testing (AST) profiles were determined using disk diffusion on Mueller-Hinton agar (Oxoid) following US Clinical and Laboratory Standards Institute (CLSI) guidelines (M100, 28 ^th^ edition, January 2018). The organisms identified were as follows: *Proteus vulgaris,* susceptible to co-amoxiclav, ciprofloxacin, gentamicin, ceftriaxone and co-trimoxazole but resistant to ampicillin; *Aeromonas sobria,* susceptible to chloramphenicol, ciprofloxacin, gentamicin, ceftriaxone, co-trimoxazole and intermediate to co-amoxiclav; ESBL-positive *Escherichia coli,* susceptible to amikacin, chloramphenicol, ciprofloxacin, gentamicin, meropenem, intermediate to co-amoxiclav and resistant to ampicillin, ceftriaxone, co-trimoxazole and tetracycline and confirmed as an ESBL producer using the double-disk method (cefotaxime +/- clavulanate and ceftazidime +/- clavulanate [BD]); *Edwardsiella tarda,* susceptible to ampicillin, co-amoxiclav, ciprofloxacin, gentamicin, ceftriaxone and co-trimoxazole; and a probable *Burkholderia* species, susceptible to co-amoxiclav, ceftazidime, meropenem and co-trimoxazole was also identified. The *Burkholderia sp*. isolate grew on Ashdown’s agar, was oxidase positive and *B. pseudomallei*-specific latex agglutination positive (Faculty of Tropical Medicine Mahidol University, Thailand), however the API 20NE gave a low result of 50.7% for *B. pseudomallei* (profile 1157577). Due to this low percentage identification, the isolate had molecular confirmation testing by PCR with a *Burkholderia* species panel which included *B. pseudomallei, B. thailandensis* and *B. cepacia* targeting a Tat domain protein, 70-kDa protein and a conserved 12-kDa protein respectively using an adapted previously published method ^8^ and was identified as *B. thailandensis*. The isolate was then referred to the Mahidol Oxford Research Unit (MORU) in Bangkok where the latex agglutination test was confirmed as positive and the isolate was then identified by MALDI-TOF (Bruker Daltonik GmbH) as *B. thailandensis* using a recently constructed library ^9^. The latex agglutination test (latex beads coated with 4B11 monoclonal antibody specific for the 200kDa exo-polysaccharide of *B. pseudomallei*) is used to rapidly identify suspect *B. pseudomallei* colonies in the Mahosot Hospital Microbiology Laboratory.

DNA was extracted from the isolate using QIAamp DNA Mini Kit (Catalogue number 56304, Qiagen, Germany), then processed for the 150-base-read library preparation and sequenced by Illumina NovaSeq 6000 system with paired-end runs at the Center for Medical Genomics, Faculty of Medicine, Ramathibodi Hospital, Bangkok, Thailand. FastQC v.0.11.9 ( https://github.com/s-andrews/FastQC) was used to pre-process sequenced reads. Raw reads were *de novo* assembled using SPAdes v3.13.1 ( https://github.com/ablab/spades). The species was identified using fIDBAC ^10^ and confirmed using FastANI v.1.31 ^11^ against *B. pseudomallei* K96243, *B. thailandensis* E264, *B. thailandensis* FDAARGOS_238 and *B. thailandensis* E555.

Seven candidate housekeeping genes ( *lepA, gmhD, nhd, lipA, nark, gltB, ace, nhd*) were selected using the sequences of *B. pseudomallei* K96243. The sequences of each gene were retrieved from the clinical isolate using Basic Local Alignment Search Tool (BLAST). Multi-locus sequence typing (MLST) was identified using *B. pseudomallei* MLST website ( https://pubmlst.org/bpseudomallei/).

**Figure 1.**
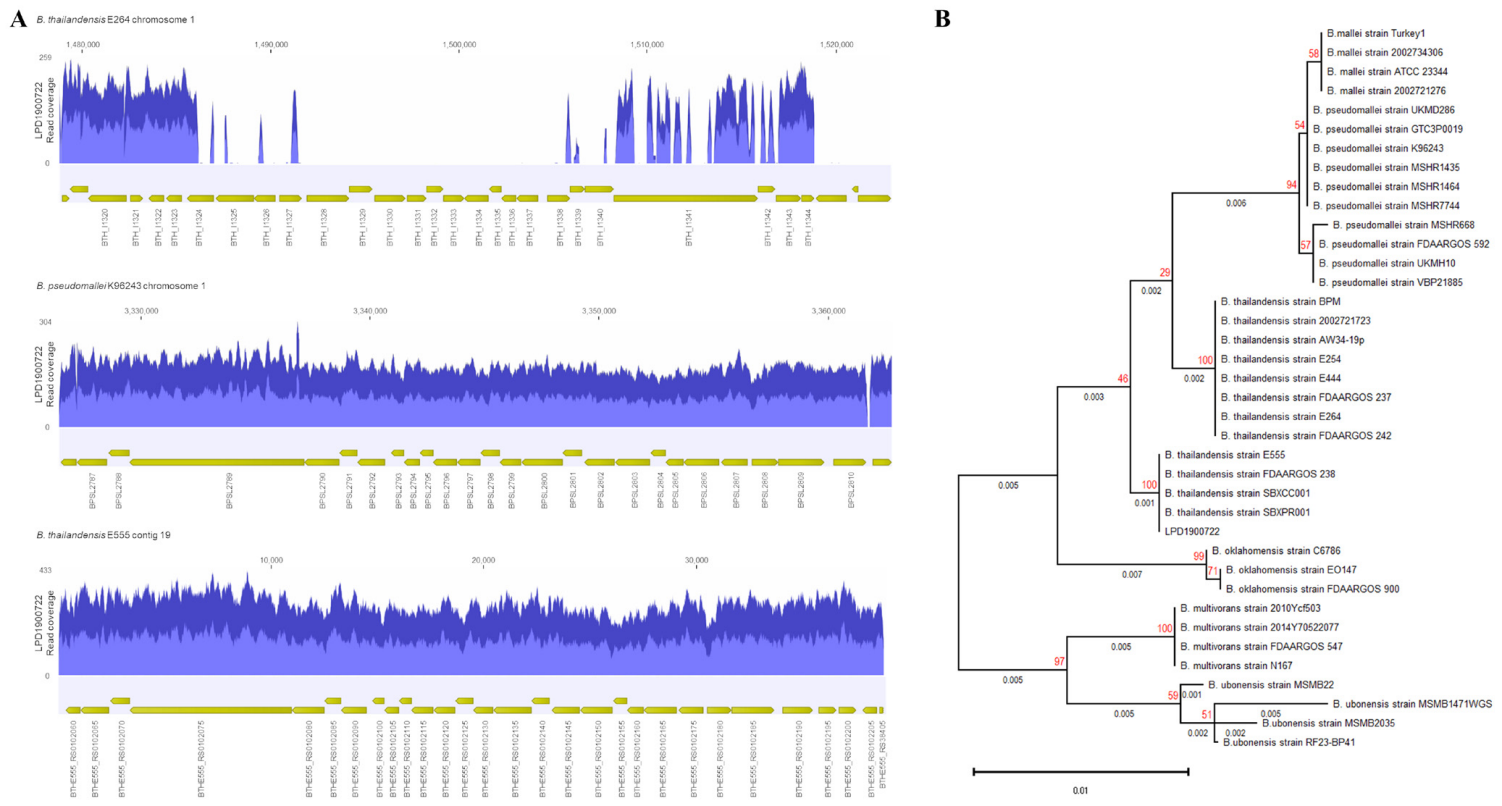
( **A**) Read coverage of LPD1900722 mapped to capsular polysaccharide gene cluster of *B. thailandensis* E264 (top), *B. pseudomallei* K96243 (middle) and *B. thailandensis* E555 (bottom). ( **B**) Phylogenetic tree analysis of full-length of 16s ribosomal *rna* of *Burkholderia* spp. and clinical isolate (LPD1900722) using a maximum-likelihood with 100 bootstraps. Number on the branches indicate branch length (black) and bootstrap values (red).

The isolate was identified using assembled genome sequence data. When submitting the genome sequences to the database through fIDBAC ^10^, the whole genome Average Nucleotide Identity (ANI) of the isolate was 98.97% compared to *B. thailandensis* E264. The species was then confirmed by incorporating *B. pseudomallei* K96243, *B. thailandensis* FDAARGOS_238 and *B. thailandensis* E555. The isolate shared only 92.95% compared to *B. pseudomallei* K96243, while the ANI was 99.72% and 99.78% when compared to *B. thailandensis* FDAARGOS_238 and *B. thailandensis* E555, respectively. *B. thailandensis* E555 is a strain which expresses a *B. pseudomallei*-like capsular polysaccharide (BTCV). The MLST analysis revealed that the patient strain (LPD1900722) shared the same sequence type (ST696) as *B. thailandensis* strains E555, but was different from E264 (ST80). While the capsule gene cluster was absent in *B. thailandensis* E264, it was present in LPD1900722, similar to the configuration found in *B. pseudomallei* K96243 and *B. thailandensis* E555 ( Figure 1A). A maximum-likelihood phylogeny tree was constructed based on complete 16S ribosomal RNA (BTH_RS17800 of *B. thailandensis* E264) which showed the clinical isolate grouping within the *B. thailandensis* clade ( Figure 1B).

## Discussion

*B. thailandensis* is a Gram negative bacterium that is closely related to *B. pseudomallei,* the causative agent of melioidosis. *B. thailandensis* is widely present in Southeast Asia and Northern Australia but has also been detected in North America and West Africa ^3, 12^. *B. thailandensis* is usually considered non-pathogenic and is commonly found in the environment (surface water and soil) in tropical or subtropical climates ^13^. *B. thailandensis* was first discovered in Thailand in 1996 and is differentiated from *B. pseudomallei* phenotypically by its ability to assimilate arabinose and it was classified as a new species in 1998 ^14, 15^. BTCV produce a capsular polysaccharide that cross-reacts with that of *B. pseudomallei*, giving rise to the potential for confusion in the laboratory ^6^. Occasional cases of infection with *B. thailandensis* have been reported in the United States, China, Thailand and Malaysia ^4, 5, 16– 19^.

As *B. thailandensis* is found in the environment, and the patient pus sample yielded a very mixed culture result, it is difficult to comment on the clinical significance of the BTCV isolate. However, this is the first documented isolation of *B. thailandensis* from a clinical specimen in Laos, and the first report of the isolation of BTCV from a clinical sample of which we are aware. The potential for confusion with *B. pseudomallei* is significant and is something of which laboratory staff should be aware to avoid incorrectly labelling a patient as having melioidosis. There have been no further *B. thailandensis* isolates identified from the Mahosot Hospital Microbiology laboratory since this isolate (up to May 2024). The isolate was initially mis-identified by latex agglutination, but subsequently confirmed to be *B. thailandensis* by PCR and MALDI-TOF having failed to identify as *B. pseudomallei* by API 20NE. Although the pathogenicity of the BTCV within this mixed infection was questionable, this undoubtedly had the potential to lead to misdiagnosis meaning that the patient could have ended up being treated unnecessarily for melioidosis, involving antibiotic treatment for more than 3 months. 

## Conclusion

This is the first case of *B. thailandensis* reported in Laos and the first report of infection with BTCV of which we are aware. Laboratory staff in melioidosis-endemic areas should be aware of the possibility of *B. thailandensis* and should ensure that appropriate confirmatory methods are used to differentiate between *B. pseudomallei* and *B. thailandensis* rather than relying on latex agglutination or other serological methods alone. 

## Public and patient involvement

There was no formal patient or public involvement in the design or conduct of this work.

## Ethics and consent

Written informed consent for publication of their clinical details was obtained.

## Data Availability

All data underlying the results are available as part of the article and no additional source data are required.
