# Supplementary material for: Case Report: Soft tissue infection with Burkholderia thailandensis capsular variant: case report from the Lao PDR
Source: Wellcome Open Res. 2024 Nov 28;9:421. Originally published 2024 Aug 1. [Version 3] doi: 10.12688/wellcomeopenres.22706.3 (PMC11377925; doi:10.12688/wellcomeopenres.22706.3)
Supplement: Peer review history

# Reviewer report: Reviewer response for version 3

**DOI:** 10.21956/wellcomeopenres.25902.r117093

**Published:** 2025-02-04

**Authors:** Enoka Corea

**Affiliations:** 1 University of Colombo, Colombo, Western Province, Sri Lanka

1. In the Background in the abstract it is stated that "Although B. thailandensis has been identified in environmental samples ... there have not been any human cases reported" This implies that there have never been previous infections with B. thailandensis anywhere in the world. Please correct to "there have not been any human cases reported in Laos"

 2. In the abstract it is better to state "A deep pus samples was taken and the patient was started on cloxacillin, ceftriaxone and metronidazole. Melioidosis was suspected from preliminary laboratory test and the treatment was then changed to ceftazidime following local melioidosis treatment guidelines"

 3. If it is not confirmed that BTCV was the actual pathogen that caused the infection the title of the article is misleading. It is better to say mixed infection including BTCV or possible infection

 4. It would be good to give the details of the preliminary pus results that led to a suspicion of melioidosis and change of treatment

Are enough details provided of any physical examination and diagnostic tests, treatment given and outcomes?

Partly

Is the case presented with sufficient detail to be useful for other practitioners?

Yes

Is sufficient discussion included of the importance of the findings and their relevance to future understanding of disease processes, diagnosis or treatment?

Yes

Is the background of the case’s history and progression described in sufficient detail?

Yes

Reviewer Expertise:

Melioidosis and antimicrobial resistance

I confirm that I have read this submission and believe that I have an appropriate level of expertise to confirm that it is of an acceptable scientific standard.

# Reviewer report: Reviewer response for version 3

**DOI:** 10.21956/wellcomeopenres.25902.r116835

**Published:** 2025-01-20

**Authors:** Himali S Jayasinghearachchi

**Affiliations:** 1 Kotelawala Defence University, Dehiwala-Mount Lavinia, Sri Lanka

The case report is well-organized, providing a clear and detailed overview of the case and its significance. This study is particularly intriguing as it identifies a BTCV-expressing *Burkholderia thailandensis* strain, which highlights the complexity of the *B. thailandensis* species and its potential clinical implications. This finding underscores the novel aspect of the case, raising the possibility that similar strains may exist in other regions.

 The authors effectively detail the patient's occupational exposure, linking it to the environmental risk factors for infection. They also address the diagnostic challenges faced in distinguishing between *B. thailandensis,* *B. pseudomallei,* and other related species. This underscores the need for the development of more refined diagnostic tools to improve accuracy and aid in better clinical decision-making.

 Is the background of the case’s history and progression described in sufficient detail?

 Yes, the authors provide sufficient details about case history, the background of the case’s history providing comprehensive knowledge about the case study.

 Are enough details provided of any physical examination and diagnostic tests, treatment given, and outcomes?

 Yes

 Is sufficient discussion included of the importance of the findings and their relevance to future understanding of disease processes, diagnosis, or treatment?

 Yes

 The authors have sufficiently discussed the challenges in determining the clinical significance of BTCV strains in mixed cultures and the potential risk of misdiagnosis, particularly with the latex agglutination test. Additionally, this study paves the way for future research to explore the distribution of BTCV strains in the environment. The conclusion is clear, emphasizing the significance of the findings and the implications for improving diagnostic accuracy and understanding disease processes.

 Is the case presented with sufficient detail to be useful for other practitioners?

 Yes, the clear description of the patient's history, occupational risk factors, and diagnostic challenges makes the case highly useful for practitioners, particularly those working in endemic regions or handling similar diagnostic uncertainties.

 Competing Interests

 No competing interests were disclosed.

 Reviewer Expertise

 Medical Microbiology, genomic epidemiology of medically important bacteria, with a focus on environmental and clinical diagnostics of *Burkholderia* species

Are enough details provided of any physical examination and diagnostic tests, treatment given and outcomes?

Yes

Is the case presented with sufficient detail to be useful for other practitioners?

Yes

Is sufficient discussion included of the importance of the findings and their relevance to future understanding of disease processes, diagnosis or treatment?

Yes

Is the background of the case’s history and progression described in sufficient detail?

Yes

Reviewer Expertise:

Burkholderia pseudomallei genomic epidemiology

I confirm that I have read this submission and believe that I have an appropriate level of expertise to confirm that it is of an acceptable scientific standard.

# Reviewer report: Reviewer response for version 3

**DOI:** 10.21956/wellcomeopenres.25902.r113704

**Published:** 2024-12-02

**Authors:** Revathy Arushothy

**Affiliations:** 1 Bacteriology Unit, Institute for Medical Research, National Institutes of Health,, Institute for Medical Research, SHAH ALAM, Selangor, Malaysia

The case report is well written. It would benefitted the readers if the authors had extensively discussed the  co-infection with other pathogens especially the ESBL E.coli.

Are enough details provided of any physical examination and diagnostic tests, treatment given and outcomes?

Yes

Is the case presented with sufficient detail to be useful for other practitioners?

Yes

Is sufficient discussion included of the importance of the findings and their relevance to future understanding of disease processes, diagnosis or treatment?

Yes

Is the background of the case’s history and progression described in sufficient detail?

Yes

Reviewer Expertise:

Bacteriology, Epidemiology

I confirm that I have read this submission and believe that I have an appropriate level of expertise to confirm that it is of an acceptable scientific standard.

# Reviewer report: Reviewer response for version 2

**DOI:** 10.21956/wellcomeopenres.25442.r111302

**Published:** 2024-11-20

**Authors:** Sylvia Chieng

**Affiliations:** 1 Universiti Kebangsaan Malaysia, Bangi, Malaysia

This article reported the first case of *B. thailandensis *in Laos, highlighting the need of appropriate diagnosis methods to distinguish between *B.pseudomallei* and *B. thailandensis*.  

 Overall, it was well-written and the case was presented clearly. No further comment from me except a spelling error in *B.thailandensis* name in the abstract.

Are enough details provided of any physical examination and diagnostic tests, treatment given and outcomes?

Yes

Is the case presented with sufficient detail to be useful for other practitioners?

Yes

Is sufficient discussion included of the importance of the findings and their relevance to future understanding of disease processes, diagnosis or treatment?

Yes

Is the background of the case’s history and progression described in sufficient detail?

Yes

Reviewer Expertise:

Bacteriology, host-pathogen interactions

I confirm that I have read this submission and believe that I have an appropriate level of expertise to confirm that it is of an acceptable scientific standard.

# Reviewer report: Reviewer response for version 2

**DOI:** 10.21956/wellcomeopenres.25442.r110287

**Published:** 2024-11-18

**Authors:** Revathy Arushothy

**Affiliations:** 1 Bacteriology Unit, Institute for Medical Research, National Institutes of Health,, Institute for Medical Research, SHAH ALAM, Selangor, Malaysia

This case description is well written and interesting. The findings, especially the detection of the BTCV in the *B. thailandensis* strain, highlight the importance of correct diagnosis in such case where an environmental bacterium is able to cause disease in humans.

 There are several suggestions:

 1. Abstract

*B. thailandensis *is spelled wrongly

**Introduction**

 May I suggest "lives in soil" to "abundant is soil" or "ubiquitous in soil"

**Case report**

 Maybe the author can explain why - despite not isolating B. pseudomallei, the treatment was changed to melioidosis treatment regime.

 Was there any further investigation performed on the ESBL -positive Escherichia coli which is also common source for soft tissue infection. 

 Was the antimicrobial susceptibility test performed for the *B. thailandensis *isolate? I suggest the authors include this information as well and possibly include in the discussion section.

Are enough details provided of any physical examination and diagnostic tests, treatment given and outcomes?

Yes

Is the case presented with sufficient detail to be useful for other practitioners?

Yes

Is sufficient discussion included of the importance of the findings and their relevance to future understanding of disease processes, diagnosis or treatment?

Yes

Is the background of the case’s history and progression described in sufficient detail?

Yes

Reviewer Expertise:

Bacteriology, Epidemiology

I confirm that I have read this submission and believe that I have an appropriate level of expertise to confirm that it is of an acceptable scientific standard.

# Reviewer report: Reviewer response for version 2

**DOI:** 10.21956/wellcomeopenres.25442.r99762

**Published:** 2024-09-30

**Authors:** Delaney Burnard

**Affiliations:** 1 University of the Sunshine Coast (Ringgold ID: 5333), Sippy Downs, Queensland, Australia; 2 Queensland Cyber Infrastructure Foundation, Saint Lucia, Queensland, Australia

I have no further comments to make.

Are enough details provided of any physical examination and diagnostic tests, treatment given and outcomes?

Yes

Is the case presented with sufficient detail to be useful for other practitioners?

Yes

Is sufficient discussion included of the importance of the findings and their relevance to future understanding of disease processes, diagnosis or treatment?

Yes

Is the background of the case’s history and progression described in sufficient detail?

Yes

Reviewer Expertise:

Bacteria, Antimicrobial Resistance, Genomics, MLST, Phylogenetics, Microbiology, Clinical Microbiology.

I confirm that I have read this submission and believe that I have an appropriate level of expertise to confirm that it is of an acceptable scientific standard.

# Reviewer report: Reviewer response for version 1

**DOI:** 10.21956/wellcomeopenres.25008.r92510

**Published:** 2024-09-05

**Authors:** Delaney Burnard

**Affiliations:** 1 University of the Sunshine Coast (Ringgold ID: 5333), Sippy Downs, Queensland, Australia; 2 Queensland Cyber Infrastructure Foundation, Saint Lucia, Queensland, Australia

Report is detailed and sound in all the above categories, with the outcome and importance of accurate laboratory diagnosis clearly conveyed.

 There are a few sentences that need to be revised to improve reading. Please see text below to be revised:

 The patient was initially started on cloxacillin, ceftriaxone and metronidazole, and was then changed to ceftazidime treatment following local melioidosis treatment guidelines. 

- Can you include the identification of *Burkholderia thailandensis *somewhere in **here, as this has been skipped completely in the abstract.

Some environmental isolates of *B. thailandensis*, including one from Lao PDR (Laos), have been shown to express a capsular polysaccharide that cross-reacts with that of *B. pseudomallei*, known as *B. thailandensis* capsular variants (BTCV), but as yet infection

-  with such a strain has not been reported ^6^.
-   do you mean "but as of yet"?

The MLST analysis revealed that the patient strain (LPD1900722) shared the same sequence type (ST696) as *B. thailandensis* strains E555, but was different from E264 (ST80). 

- "differing from strain E264 (ST80)"

The potential for confusion with *B. pseudomallei* is significant and is something of which laboratory staff should be aware to avoid incorrectly labelling a patient as having melioidosis.

- "should be aware of"

I would also like to ask the authors why they chose to provide a 16S phylogeny over a whole genome, cgMLST or MLST phylogeny. Given the authors performed typing on the isolate and further describe relatedness of strains using MLST, I expected to see a *B. thailandensis* MLST phylogeny. I think the paper would be more robust, provide greater insight into the relatedness of strains and align with the manuscript text better with a MLST phylogeny in Figure 1.

Are enough details provided of any physical examination and diagnostic tests, treatment given and outcomes?

Yes

Is the case presented with sufficient detail to be useful for other practitioners?

Yes

Is sufficient discussion included of the importance of the findings and their relevance to future understanding of disease processes, diagnosis or treatment?

Yes

Is the background of the case’s history and progression described in sufficient detail?

Yes

Reviewer Expertise:

Bacteria, Antimicrobial Resistance, Genomics, MLST, Phylogenetics, Microbiology, Clinical Microbiology.

I confirm that I have read this submission and believe that I have an appropriate level of expertise to confirm that it is of an acceptable scientific standard, however I have significant reservations, as outlined above.

# Response

**Published:** 2024-09-09

**Authors:** Tamalee Roberts

**Affiliations:** Microbiology, Lao-Oxford-Mahosot Hospital Wellcome Trust Research Unit, Vientiane, Vientiane Capital, Lao People's Democratic Republic

The authors would like to thank the reviewer for their comments. Please find the specific replies to comments below.

**Reviewer comment:** Can you include the identification of Burkholderia thailandensis somewhere in here, as this has been skipped completely in the abstract.

**Response: **Thank you for the comment. The patient was changed to ceftazdime before a diagnosis of *B. thailandensis *was made.

**Reviewer comment: **Some environmental isolates of B. thailandensis, including one from Lao PDR (Laos), have been shown to express a capsular polysaccharide that cross-reacts with that of B. pseudomallei, known as B. thailandensis capsular variants (BTCV), but as yet infection with such a strain has not been reported.

  Do you mean "but as of yet"?

**Response: **Thank you for pointing this out. This has been updated. **Reviewer comment: **The MLST analysis revealed that the patient strain (LPD1900722) shared the same sequence type (ST696) as B. thailandensis strains E555, but was different from E264 (ST80).  "differing from strain E264 (ST80)"

**Response: **This has been changed.

**Reviewer comment: **The potential for confusion with B. pseudomallei is significant and is something of which laboratory staff should be aware to avoid incorrectly labelling a patient as having melioidosis. "should be aware of"

**Response: **​​​​​​​Thank you for highlighting this. ‘of’ has been added.

**Reviewer comment: **I would also like to ask the authors why they chose to provide a 16S phylogeny over a whole genome, cgMLST or MLST phylogeny. Given the authors performed typing on the isolate and further describe relatedness of strains using MLST, I expected to see a B. thailandensis MLST phylogeny. I think the paper would be more robust, provide greater insight into the relatedness of strains and align with the manuscript text better with a MLST phylogeny in Figure 1.

**Response: **Thank you for your valuable suggestions. We fully acknowledge the significance of performing a whole-genome or MLST phylogeny to gain a more comprehensive understanding of the relatedness between *Burkholderia thailandensis* strains. In response, we are planning a separate, more detailed study where we will expand our dataset to encompass all available *B. thailandensis* genomes. This will allow us to conduct an in-depth genomic analysis, which will provide a more robust comparison of strain relationships, including the identification of virulence genes and their geographic distribution. This study serves as a foundation for future analyses. We appreciate your understanding of the study’s scope and look forward to exploring these important aspects in the future.

# Reviewer report: Reviewer response for version 1

**DOI:** 10.21956/wellcomeopenres.25008.r92503

**Published:** 2024-09-05

**Authors:** Mohd Firdaus Raih

**Affiliations:** 1 Universiti Kebangsaan Malaysia, Bangi, Malaysia

Increasing reports of *B. thailandensis* infections is an important contribution to the field in order to highlight the threat also posed by *B. thailandensis* which was previously thought to be less or even non-infectious.

 The manuscript is clearly written and all the elements required of a case report is presented.

Are enough details provided of any physical examination and diagnostic tests, treatment given and outcomes?

Yes

Is the case presented with sufficient detail to be useful for other practitioners?

Yes

Is sufficient discussion included of the importance of the findings and their relevance to future understanding of disease processes, diagnosis or treatment?

Yes

Is the background of the case’s history and progression described in sufficient detail?

Yes

Reviewer Expertise:

Bacterial pathogenesis, bacterial genomics

I confirm that I have read this submission and believe that I have an appropriate level of expertise to confirm that it is of an acceptable scientific standard.

# Response

**Published:** 2024-09-09

**Authors:** Tamalee Roberts

**Affiliations:** Microbiology, Lao-Oxford-Mahosot Hospital Wellcome Trust Research Unit, Vientiane, Vientiane Capital, Lao People's Democratic Republic

The authors thank the reviewer for their supportive comments.

# Reviewer report: Reviewer response for version 1

**DOI:** 10.21956/wellcomeopenres.25008.r92509

**Published:** 2024-08-31

**Authors:** Abdel Rahman Zueter

**Affiliations:** 1 The Hashemite University, Zarqa, Jordan

The case description is concise and to the point. It provides essential clinical details, including the patient's symptoms, physical examination findings, and initial treatment. The progression from preliminary suspicion of melioidosis to the adjustment of treatment according to local guidelines is logical and well-explained.

 Since the case is very rare, it is good to add a table to review all similar cases 

Are enough details provided of any physical examination and diagnostic tests, treatment given and outcomes?

Yes

Is the case presented with sufficient detail to be useful for other practitioners?

Yes

Is sufficient discussion included of the importance of the findings and their relevance to future understanding of disease processes, diagnosis or treatment?

Yes

Is the background of the case’s history and progression described in sufficient detail?

Yes

Reviewer Expertise:

Interested in Laboratory Medicine and Infectious Diseases. Did and joined several helminthology and bacteriology projects and case reports. Current area of expertise include Clinical epidemiology, MLST-based molecular epidemiology; tropical infections, and laboratory diagnostics.

I confirm that I have read this submission and believe that I have an appropriate level of expertise to confirm that it is of an acceptable scientific standard.

# Response

**Published:** 2024-09-09

**Authors:** Tamalee Roberts

**Affiliations:** Microbiology, Lao-Oxford-Mahosot Hospital Wellcome Trust Research Unit, Vientiane, Vientiane Capital, Lao People's Democratic Republic

We are grateful to the reviewer for their thoughtful reading and comments. This is the first human case of BTCV of which we are aware of and therefore there are no similar cases.
